# Influence on Depression, Anxiety, and Satisfaction of the Relatives' Visit to Intensive Care Units prior to Hospital Admission for Elective Cardiac Surgery: A Randomized Clinical Trial

**DOI:** 10.1155/2022/1746782

**Published:** 2022-05-02

**Authors:** Sara González-Martín, Ricardo Becerro-de-Bengoa-Vallejo, Moisés Rodríguez-García, Marta Elena Losa-Iglesias, Victoria Mazoteras-Pardo, Patricia Palomo-López, David Rodríguez-Sanz, César Calvo-Lobo, Daniel López-López

**Affiliations:** ^1^Faculty of Nursing, Physiotherapy and Podiatry, Universidad Complutense de Madrid, Madrid, Spain; ^2^Faculty of Health Sciences, Universidad Rey Juan Carlos, Móstoles, Spain; ^3^Department of Nursing, Physiotherapy and Occupational Therapy, School of Physiotherapy and Nursing, University of Castilla-La Mancha, Toledo, Spain; ^4^University Center of Plasencia, Universidad de Extremadura, Plasencia, Spain; ^5^Research, Health and Podiatry Group, Department of Health Sciences, Faculty of Nursing and Podiatry, Industrial Campus of Ferrol, Universidade da Coruña, A Coruña, Spain

## Abstract

**Background:**

Intensive care units (ICUs) may produce stress on the relatives of patients that have long-term physiological and psychological implications.

**Objectives:**

This study aimed to evaluate the effects of the relatives´ visit prior to hospital admission(s) on the patient's scheduled cardiac surgery regarding depression, anxiety, and satisfaction of the patient's family in an ICU.

**Methods:**

A randomized clinical trial [NCT03605420] was carried out according to the CONSORT criteria. Thirty-eight relatives of ICU patients were recruited at an ICU and randomized into study groups. Experimental group participants (*n* = 19) consisted of relatives who received 1 ICU visit prior to the patient's admission. Control group participants (*n* = 19) consisted of patients' relatives who received standard care alone. A self-report test battery, including the Impact of Event Scale-Revised (IES-R) and the Hospital Anxiety and Depression Scale (HADS), was completed by the patient's relative prior to the patient's ICU admission and again three and 90 days after ICU discharge. Furthermore, the Family Satisfaction with Care in the Intensive Care Unit (FS-ICU) and Critical Care Family Needs Inventory (CCFNI) were administered to help determine the respondents' satisfaction three days after the patient's ICU discharge.

**Results:**

Statistically significant differences in FS-ICU results were found between control and experimental groups; no statistically significant differences were found in IES-R, HADS, and CCFNI results. Thus, members in the control group were more satisfied with the time elapsed to raise their concerns (*p*=0.005), emotional support provided (*p*=0.020), quality of care (*p*=0.035), opportunities to express concerns and ask questions (*p*=0.005), and general satisfaction with the ICU's decision-making (*p*=0.003).

**Conclusions:**

Relatives' satisfaction during patients' ICU admission may be impaired after their prior visit to the hospital admission. Relative's anxiety and depression scores did not seem to be significantly affected. Relatives´ visit prior to elective cardiac surgery hospital admission impaired their satisfaction in an ICU and may not be advisable for healthcare practice.

## 1. Introduction

Intensive care units (ICUs) may be considered a health service for critical patients reporting a high economic burden [1]. Certainly, this health service may be considered stressful in the long term for critical patients who suffer from physical and psychological conditions [2]. Multiple causes have been proposed as possible reasons for these alterations. Multiple potential triggers have been identified including invasive procedures, separation from relatives, lack of mobility and privacy, soreness, mechanical ventilation necessities, noise pollution, poor orientation, sleep disturbances, and lack of familiarity with nursing or medical staff [3–5]. Doubtlessly, relatives of patients in ICUs should be informed about the ICU's procedures, protocols, intervention routines, and day-to-day operations by nurses and medical doctors in order to provide optimal care. Therefore, nurses may play a main role to provide information to ICU patients on critical care necessities [6].

In addition, a high proportion of relatives of ICU patients suffered from anxiety (70%) and depression (35%), with acute and posttraumatic stress being the most common conditions. Effective communication strategies may be considered as key interventions to improve the aforementioned conditions using an easily understood language from medical team members [7]. Worldwide, different questionnaires have been used to assess depression and anxiety in relatives of ICU patients, which may easily be administered by nursing staff without formal training [8]. Furthermore, specific questionnaires such as the Hospital Anxiety and Depression Scale (HADS) [9, 10] and the Impact of Event Scale-Revised (IES-R) [11, 12] were considered adequate tools to measure depression and anxiety symptomatology as well as discomfort, in relatives of ICU patients, respectively.

Up to 69.1% of families of ICU patients may experience anxiety or stress [13], resulting in long-term secondary psychological and physiological effects [14]. Thus, other questionnaires may be useful to evaluate the relative's satisfaction during the patient's ICU admission, such as the Critical Care Family Need Inventory (CCFNI) [15, 16] and the Family Satisfaction with Care in the Intensive Care Unit (FS-ICU) [17].

Stress, depression, and/or anxiety experienced by an ICU patient's relative may be triggered by multiple secondary stressors presented in ICU environments [3–5]. Indeed, relatives of ICU patients may suffer from these symptoms even more than patients [14], with elective cardiac surgery being considered a common, controlled, and scheduled procedure to carry out the present study in an ICU environment. The effect of a preadmission intervention by the visit of patients' relatives to the ICU has not yet been explored. We hypothesized that the visit of patients' relatives to the ICU by a preadmission program could reduce depression and anxiety levels and increase satisfaction in intervention group participants. Consequently, this study aimed to evaluate the effects of a preadmission ICU visit on negative psychological symptoms and the satisfaction of patients' relatives prior to elective cardiac surgery.

## 2. Methods

### 2.1. Design

This study was a parallel, randomized controlled clinical trial to measure depression, anxiety, and satisfaction in ICU patients' relatives who have or have not received an ICU visit prior to hospital admission. This trial was carried out according to the Consolidated Standards of Reporting Trials guidelines [18]. This research was approved by the Ethical Committee of the University Hospital of La Princesa (Spain). This protocol was registered at Clinicaltrials.gov [NCT03605420].

### 2.2. Recruitment and Participants

A total of 38 participants were recruited during the patients' admission for elective cardiac surgery at the University Hospital of La Princesa (Spain). Relatives were received the day before the surgery together with the patient. A nurse of the research group recruited all participants, checking the surgical schedule of each week. Relatives were recruited from August 22, 2018, to January 25, 2019. Fortunately, all relatives (*n* = 38) of ICU patients, who were included in the study, completed the study course. Inclusion criteria comprised relatives of patients undergoing a scheduled surgery who endorsed a familial and/or sentimental relationship with the patient (i.e., a partner, parent, sibling, child, or friend), providing consent for voluntary participation, aged over 18 years, and speaking the Spanish language. Exclusion criteria consisted of relatives with impaired cognition or communication limitations, and relatives of patients who were readmitted to the ICU secondary to the deterioration [8].

### 2.3. Medical-Surgical ICU Characteristics

In the present study, the ICU presented 22 beds. In order to properly perform the research procedure, only patients with a scheduled cardiac surgery were included. Neither cardiac surgeons, nurses, patients, nor relatives preprocedurally knew whether they were involved in the study. Therefore, the information provided by surgeons and nurses did not influence the study results due to all patients received the same information regardless of whether they participated in the study [19].

### 2.4. Randomization Procedure and Intervention Protocol

Microsoft Excel was used to generate random numbers in order to assign relatives (who were codified by a number) to either the intervention or control groups (which were codified by a letter) by allocation concealment. Assignment to each group was performed exclusively by a nursing staff member (who was not involved in the research) on the day before the surgery. This procedure avoided the influence on the relatives' answers in addition to the ICU team was different for the intervention and outcome measurements. In the intervention group, a nurse explained a normal ICU room composition and functioning and the routine scheduled with this type of intervention (i.e., surgery procedure, eating schedule, staff who was involved in the patient's care, and visiting schedule). In the control group, all participants received the standardized set of information provided to all new ICU patients on the day of the surgery and after the procedure. This information was presented as a brochure which contained the medical visiting schedules and the patients' necessities in an ICU, such as a toothbrush, house slippers, moisturizer, radio, or books. A stratified randomization protocol was used, depending on the ICU admission week, in order to avoid the coincidence of the relatives of control and intervention groups in the same hospitalization or waiting room prior to the intervention. At the hospital, surgeons attended from Monday to Friday. Surgeons did not contact the patients since they signed the consent until the surgery day in the operating room. Thus, surgeons did not know the study group allocation. ICU staff was composed of professionals specialized in cardiac and traumatic patients and personnel specialized in neurocritical care. The neurocritical staff received the participants without knowing the study group allocation.

Usually, patients' relatives who attended surgery in the same weeks shared the same waiting rooms while they stayed in the ICU. In addition, patients who stayed in hospitalization usually shared the same room with another patient with a similar diagnosis and were admitted to the hospital on the same days. This fact was the reason which explained the randomization procedure depending on the week of ICU admission.

Experimental group participants (*n* = 19) consisted of relatives who received 1 ICU visit prior to the patient's admission. Control group participants (*n* = 19) consisted of patients' relatives who received standard care alone [19].

### 2.5. Descriptive Data

The following descriptive data were collected from patients' relatives: sex, age, weight, height, body mass index (BMI), educational level, religious beliefs, prior psychiatric conditions, prior ICU experiences as a relative or patient according to the self-report, daily frequency of visits to the patient, biological relationship to the patient, qualitative relationship with the patient in daily life, proximity to the hospital, and decision-making. The following descriptive data were collected on ICU patients: duration of ICU stay (days), duration of mechanical ventilation (days), duration of ICU benzodiazepine use (days), reintervention (i.e., patients requiring a second surgery following a problematic first surgery), use of renal replacement therapy, use of blood transfusion, reintubation, surgical complications, mortality, delirium episodes, and physical sequelae [19].

### 2.6. Outcome Measures and Follow-Up

The IES-R [9, 11, 12] and HADS [20–23] instruments were self-report measures and were completed by the relatives of ICU patients prior to admission (more specifically, before the experimental or control interventions, preprocedurally). Instruments were completed by all participants at time 1 (baseline), time 2 (3 days postdischarge), and time 3 (90 days postdischarge). Both tools were used 90 days after ICU discharge according to a diagnosis of posttraumatic stress syndrome and three days after discharge according to a prior study evaluating these symptoms in ICUs [24]. Furthermore, the FS-ICU [17] and CCFNI [15, 16] were administered to assess the relatives' satisfaction three days after discharge from the ICU.

### 2.7. Hospital Anxiety and Depression Scale (HADS)

The HADS assessed the anxiety and depression levels in relatives of hospitalized and nonpsychiatric patients [20–23]. This self-administered questionnaire comprised 14 items divided into two subgroups of seven items; one subgroup assessed depression and another group evaluated anxiety. Each item was scored on a Likert-type scale ranging from 0 to 3. Regarding the anxiety subscale, questions were focused on psychic symptoms, such as tension, nervousness, apprehension, worry, uneasiness, nervousness, or anguish, and corresponded to the odd-numbered questions. According to the depression subscale, questions were focused on anhedonia, such as enjoyment, joy, dullness, interest in personal appearance, or illusion, and corresponded to the even-numbered items. Both subscales presented a score ranging from 0 to 21 points. This tool was validated in the Spanish language and Cronbach's *α* coefficients showed an adequate internal consistency for the total score (0.90), the depression subscale (0.84), and the anxiety subscale (0.85) [10]. Furthermore, good reliability was shown by factorial analysis for the anxiety subscale (0.80) and the depression subscale (0.85). Cut-off scores were used to divide the total score of each subscale into three levels of significance, namely, normal (0–7 points), possible or uncertain (8–10 points), and probable or affirmative case of anxiety or depression (11–21 points) [25].

### 2.8. Impact of Event Scale-Revised (IES-R)

The IES-R was used to determine the subjective discomfort accompanying stressful and/or traumatic experiences of relatives of ICU patients. This self-administered questionnaire comprised 22 items assessing the relatives' experiences during the past week (scored on a Likert-type scale from 0 to 3), which was divided into three subscales: intrusion, avoidance, and hyperexcitation [9, 11, 12]. Cronbach's *α* coefficients showed an adequate internal consistency for intrusion (0.87), hyperexcitation (0.79), and avoidance (0.85), with good test–retest reliability coefficients for each domain (0.57, 0.59, and 0.51, respectively) [11]. Cronbach's *α* coefficients of this Spanish-validated tool showed an adequate internal consistency for the total score (0.86), the intrusion subscale (0.78), and the avoidance subscale (0.82). Nevertheless, the hyperexcitation subscale showed some sex discrepancies in the total sample (0.19), and for men (0.10) but not for women (0.80) [12].

### 2.9. Critical Care Family Need Inventory (CCFNI)

The CCFNI was used to assess the perceived needs of relatives of ICU patients. This self-administered scale comprised 45 items (scored on a Likert-type scale from 1 to 4) divided into four subscales: medical care, personal attention, communication, and perceived improvements. Cronbach's *α* coefficients showed an adequate internal consistency for the total score (0.65), medical care (0.60), communication (0.60), personal attention (0.60), and perceived improvements (0.64). Correlation coefficients of each item with respect to the total score were greater than 0.30, with a correct homogeneity index [15, 16].

### 2.10. Family Satisfaction with Care in Intensive Care Unit (FS-ICU)

This scale was applied to determine the relatives' satisfaction with the ICU's services. FS-ICU was considered as a self-administered questionnaire comprising 35 items (scored on a Likert-type scale from 1 to 5) divided into two subscales: healthcare satisfaction and decision-making satisfaction. Furthermore, this tool contained three open-ended questions (i.e., areas of improvement, items to point out well done, and important items not mentioned) in which common groups were identified (i.e., noise levels and bathroom accessibility) [17]. Cronbach's *α* coefficients showed an adequate internal consistency for the total score (0.84) and the subscales (0.74–0.97); a correlation of 0.63 attested to adequate psychometric properties [26].

### 2.11. Calculation of the Sample Size

Sample size calculation was determined by the GRANMO software (v.7.11) in line with prior research that assessed an education protocol for the relatives of ICU patients using the CCFNI as the primary outcome measure [24] and considering our ICU characteristics, including a 22-bed service with 5–7 surgeries per week. Scores of 145.58 ± 15.91 in the experimental group and 132.05 ± 13.55 in the control group were considered providing an effect size of *d* = 0.91. Furthermore, two-tailed analyses, *α* error = 0.05, desired power = 80% (*β* = 20%), and possible loss = 10% were used for sample size calculation. Consequently, a total sample of 38 participants was determined (19 per experimental condition).

### 2.12. Statistical Analysis

All statistical analyses were performed with the “IBM SPSS Statistics” software (v.22.0, SPSS Inc., Chicago, IL). *p* value < 0.05 was considered statistically significant for a confidence interval (CI) of 95%. The Shapiro–Wilk test was applied to determine normality. Considering the quantitative data, the Wilcoxon test for related samples and the Student's *t*-test for paired samples were applied to compare findings among follow-up periods for nonparametric and parametric data, respectively. The Mann–Whitney *U* test and the independent Student's *t*-test were also applied to compare findings between the experimental and control group. Furthermore, repeated-measures general linear model (GLM) analysis was performed to compare intrasubject factors within and between the HADS and IER-S scales, and post hoc analyses were conducted according to Bonferroni's correction adjustments. Regarding the categorical data, Chi-square tests were used to determine differences between the experimental and control group [27]. Finally, intention-to-treat analysis was considered for all statistical calculations.

## 3. Results

### 3.1. Descriptive Features

**Figure 1:**
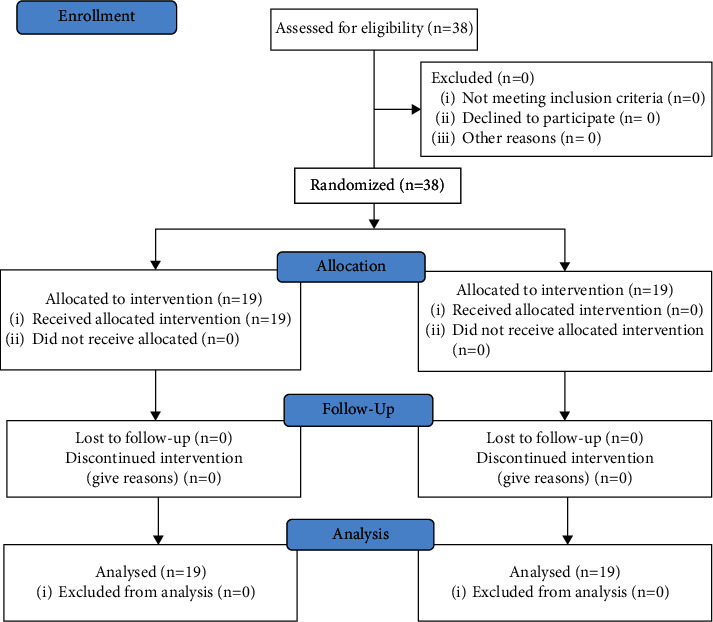
Flow diagram.

From a total sample of 40 participants initially assessed for eligibility, 38 participants were included and completed the study (two relatives declined to participate) (Figure 1). Descriptive features did not show any statistically significant difference (*p* > 0.05) between control and experimental groups for age, weight, height, BMI, ICU stay, days of mechanical ventilation, benzodiazepines intake, sex, education level, religious beliefs, previous psychiatric illness, prior ICU experience as patient or relative, reintervention, renal replacement therapy or blood transfusions use, reintubation, patient´s deaths, delirium episodes, physical sequelae at discharge, visit number per day, familiar relationship with the patient or relationship with the patient in daily life, closeness to the hospital, and decision-making (Table 1).

### 3.2. Outcome Measures

Statistically significant differences were found between the control and experimental group for the FS-ICU. Nevertheless, statistically significant differences were not found for the HADS (Table 2), IES-R (Table 3), or CCFNI (Table 4). Thus, members in the control group were more satisfied with the time elapsed to raise their concerns (*p*=0.005), emotional support provided (*p*=0.020), quality of care (*p*=0.035), opportunities to express concerns and ask questions (*p*=0.035), and general satisfaction with the ICU's decision-making (*p*=0.003). Statistically significant differences were not found with respect to the open-ended questions (Tables 5 and 6).

## 4. Discussion

While previous studies have evaluated the effect of an open visiting policy [28] and an informative educational protocol [24] on relatives of ICU patients, the findings of the present study may provide useful information for future research on nursing management when evaluating the effects of the relatives´ visit prior to hospital admission on depression, anxiety, and satisfaction of the ICU patients' relatives. Despite the existence of multiple triggers for stress, anxiety, and/or depression of ICU patients' relatives [3–5], the effects of the ICU visit of the patients´ relatives prior to hospital admission for elective surgery did not affect depression or anxiety levels but impaired their satisfaction of the ICU's services. The relatives' visit to ICU prior to hospital admission may impair their satisfaction with the time elapsed before their concerns could be raised, emotional support offered, quality of care, opportunity to express concerns and ask questions, and general satisfaction with the ICU's decision-making. Interestingly, Khaleghparast et al. reported that 55.1% of patients and relatives were dissatisfied with the limited visiting policies in cardiac ICUs [29]. In the present study, the ICU visit by the patients' relatives prior to hospital admission did not modify their expectations. This was demonstrated by lower overall satisfaction compared to relatives with no prior hospital admissions [30].

A previous quasi-experimental study evaluated the use of an informed educational protocol on two randomized groups of patients' relatives [24]. Chien et al.‘s study was used to justify our inclusion criteria due to the fact that the study used an educational program to reduce anxiety, resulting in higher satisfaction with the care provided in the ICU. Using the same short-term follow-up of three days after discharge, the experimental group received an individualized educational program attending to the relatives' specific needs upon the patient's ICU admission, compared to a control group of relatives who received the standardized information protocol. The anxiety levels measured by the Chinese version of the State-Trait Anxiety Inventory (C-STAI) and satisfaction levels measured by the Chinese version of the CCFNI were self-reported by 66 relatives of patients admitted to ICUs. These findings conflicted with our study findings due to their intervention determined that the anxiety and depression levels reported by the relatives decreased, and that overall satisfaction levels with the ICU's services increased [24]. While long-term follow-up was not undertaken in the present study, experimental group participants demonstrated increased anxiety and depression levels (without significant differences) upon short-term follow-up, as well as lower overall ICU service satisfaction levels.

These findings were unexpected because new information could have provided the patients and relatives with more psychological control in these situations, decreasing their levels of anxiety and depression, and getting better levels of satisfaction, if the information had been provided earlier, or using other types of information (i.e., videos or photos). Some possible reasons that could explain the poor satisfaction secondary to the patient's relatives' visit prior to elective cardiac surgery hospital admission may be that expectations and views from both relatives and patients should be sought formally before ICU admission to intensive care and preoperative education should be included in addition to an ICU tour [31]. In addition, clinical outcomes of the patients were not measured in our study and patients' outcomes could play a role in decreased satisfaction of the families, in spite of the received care seems to be more related to patient's care than clinical outcomes, which could have influenced relatives' satisfaction [32]. Nevertheless, our trial did not show between-group differences for key clinical outcomes predictors in surgery such as ICU stay, days of mechanical ventilation, benzodiazepines intake, previous psychiatric illness, prior ICU experience as patient or relative, reintervention, renal replacement therapy or blood transfusions use, reintubation, patient´s deaths, delirium episodes, physical sequelae at discharge, visit number per day, familiar relationship with the patient or relationship with the patient in daily life, closeness to the hospital, and decision-making [33].

### 4.1. Implications for Healthcare Practice

According to Chien et al. [24], an individualized educational program addressing the specific needs of each relative upon the patient's ICU admission decreased depression and anxiety and increased the relatives' satisfaction with the ICU's services. While the present study was not effective in increasing relatives' satisfaction levels, a tailored educational program may be important for relatives of elective cardiac surgical patients admitted to ICU. Overall, preadmission ICU visits for patients' relatives may be not advisable due to the reduction in satisfaction levels.

Linking evidence to action, the ICU visit of relatives prior to hospital admissions appeared not to impact depression or anxiety. These participants showed lower satisfaction levels with the ICU's services with respect to time elapsed to raise concerns, emotional support, quality of care, opportunities to express concerns and pose questions, and general satisfaction with the ICU's treatment decisions. Healthcare specialists should implement an individualized educational program upon the patient's ICU admission tailored to the specific needs of each relative since this has been shown to reduce depression and anxiety symptoms and raise satisfaction levels [24]. Healthcare specialists should remain cognizant of the lowered satisfaction demonstrated by relatives who underwent visits prior to hospital admissions. Individualized educational programs for relatives should be systematically incorporated into multimodal healthcare practices.

Regarding ICU patients' relatives for elective cardiac surgery, these relatives presented similar symptoms, i.e., depression, as other relatives of other ICU patients, with elective cardiac surgery being a common and scheduled procedure that should implement novel interventions to improve the mental health of patients' relatives [34].

### 4.2. Limitations

The present study has some limitations that should be considered. Firstly, the sample was limited to relatives of cardiac patients for elective surgery. Therefore, relatives of patients who suffer from different pathologies should be investigated further to reinforce the present study's findings, given that relatives' satisfaction could vary depending on the patient's medical condition [35]. Furthermore, our research did not include any relatives of patients who died during the course of the study, which may be likely to influence depression and anxiety symptoms [36]. Regardless of the fact that statistically significant differences were not found between both groups (Table 1), and all scores were self-report measures, specific tests should be applied in future studies in order to investigate previous ICU experiences. In addition, standardized scale scores for the assessment of patient severity were not obtained, since the type of patients who undergo this elective surgery followed a similar progression [19]. Furthermore, Cronbach's alpha coefficient for the CCFNI was borderline (0.6–0.65) and other tools should be considered for future intervention studies [15, 16]. Finally, in contempt of the fact that CCFNI from a prior education protocol for relatives of ICU patients was used for sample size calculation [24], other outcome measurements such as the primary outcomes of anxiety and depression (NCT03605420) should be used for sample size calculations in future randomized clinical trials, and this has not been possible in the present study due to the lack of prior studies using these primary outcomes in relatives of ICU patients receiving a similar intervention.

## 5. Conclusions

Relatives of patients hospitalized in ICU after undergoing cardiac surgery may experience reduced satisfaction levels after attending a preadmission program a visit prior to hospital admission. Nevertheless, the relative's anxiety and depression scores did not seem to be significantly affected. Relatives´ visit prior to elective cardiac surgery hospital admission impaired their satisfaction in an ICU and may not be advisable for healthcare practice.

## Figures and Tables

**Table 1:** Descriptive features between control and experimental groups of ICU patients´ relatives.

	Control (*n* = 19)	Experimental (*n* = 19)	Control vs experimental
	Mean ± SD/median (IL-SL, 95% CI)	Mean ± SD/median (IL-SL, 95% CI)	*p*-value
Age (years)	52.42 ± 16.56/7.44 (35.86–59.86)	52.68 ± 14.78/6.64 (46.03–59.33)	0.959 ^*∗∗∗*^
Weight (Kg)	76.05 ± 14.16/6.36 (61.88–82.42)	69.94 ± 12.32/5.54 (64.40–75.48)	0.164 ^*∗∗∗*^
Height (m)	1.69 ± 0.08/0.03 (1.61–1.73)	1.63 ± 0.10/0.04 (1.58–1.67)	0.044 ^*∗∗∗*^
BMI (Kg/m^2^)	26.30 ± 4.02/1.81 (22.28–28.11)	26.22 ± 4.50/2.02 (24.19–28.24)	0.953 ^*∗∗∗*^
ICU stay (days)	8 ± 12.95/5.82 (−4.95–13.82)	4 ± 3.34/1.50 (2.49–5.50)	0.200 ^*∗∗∗*^
Mechanical ventilation (days)	3.47 ± 6.91/3.10 (−3.44–6.58)	1.63 ± 1.49/0.67 (0.95–2.30)	0.263 ^*∗∗∗*^
Benzodiazepines intake (days)	6.42 ± 13.54/6.08 (−7.12–12.50)	1.68 ± 1.52/0.68 (0.99–2.37)	0.138 ^*∗∗∗*^

	Frequency (%)	Frequency (%)	*p*-value
Sex			
Men Women	10 (52.6%) 9 (47.4%)	8 (42.1%) 11 (57.9%)	0.516 ^*∗∗*^
Education			
Without studies Primary education Secondary education High school University	2 (10.5%) 2 (10.5%) 3 (15.8%) 6 (31.6%) 6 (31.6%)	2 (10.5%) 7 (36.8%) 5 (26.3%) 2 (10.5%) 3 (15.8%)	0.179 ^*∗∗*^
Religious beliefs			
Christian Atheist Jehovah's witness Nondenominational Muslim	14 (73.7%) 1 (5.3%) 0 (0%) 4 (21.1%) 0 (0%)	13 (68.4%) 1 (5.3%) 1 (5.3%) 3 (15.8%) 1 (5.3%)	0.703 ^*∗∗*^
Previous psychiatric illness			
Yes No	1 (5.3%) 18 (94.7%)	2 (10.5%) 17 (89.5%)	0.547 ^*∗∗*^
Prior ICU experience as a patient			
Yes No	2 (10.5%) 17 (89.5%)	4 (21.1%) 15 (78.9%)	0.374 ^*∗∗*^
Prior ICU experience as a relative			
Yes No	11 (57.9%) 8 (42.1%)	7 (36.8%) 12 (63.2%)	0.194 ^*∗∗*^
Reintervention			
Yes No	4 (21.1%) 15 (78.9%)	3 (15.8%) 16 (84.2%)	0.676 ^*∗∗*^
Renal replacement therapy use			
Yes No	0 (0%) 19 (100%)	0 (0%) 19 (100%)	N/A
Use of blood transfusions			
Yes No	12 (63.2%) 7 (36.8%)	13 (68.4%) 6 (31.6%)	0.732 ^*∗∗*^
Reintubation			
Yes No	3 (15.8%) 16 (84.2%)	1 (5.3%) 18 (94.7%)	0.290 ^*∗∗*^
Patient´s death			
Yes No	0 (0%) 19 (100%)	0 (0%) 19 (100%)	N/A
Delirium episode			
Yes No	5 (26.3%) 14 (73.7%)	4 (21.1%) 15 (78.9%)	0.703 ^*∗∗*^
Physical sequelae at discharge			
Yes No	9 (47.4%) 10 (52.6%)	11 (57.9%) 8 (42.1%)	0.516 ^*∗∗*^
Visit number per day			
None Once a day Twice a day	0 (0%) 0 (0%) 19 (100%)	0 (0%) 0 (0%) 19 (100%)	N/A
Familiar relationship with the patient			
Partner Mother father Son Sister Friend	8 (42.1%) 0 (0%) 8 (42.1%) 2 (10.5%) 1 (5.3%)	9 (47.4%) 0 (0%) 10 (52.6%) 0 (0%) 0 (0%)	0.350 ^*∗∗*^
Relationship with the patient in daily life			
Daily Several times per week Weekly Monthly Annually	11 (57.9%) 3 (15.8%) 2 (10.5%) 3 (15.8%) 0 (0%)	12 (63.2%) 3 (15.8%) 3 (15.8%) 1 (5.3%) 0 (0%)	0.743 ^*∗∗*^
Closeness to the hospital			
Same town Different town	6 (31.6%) 13 (68.4%)	4 (21.1%) 15 (78.9%)	0.461 ^*∗∗*^
Decision-making			
No Isolated In group	2 (10.5%) 8 (42.1%) 9 (47.4%)	0 (0%) 11 (57.9%) 8 (42.1%)	0.282 ^*∗∗*^

SD, standard deviation; BMI, body mass index: 95% CI, 95% confidence interval; IL, inferior limit; SL, superior limit; N/A, not applicable; ICU, intensive care unit. A *p* value < 0.05 was considered statistically significant with a 95% CI. ^*∗*^Wilcoxon–Mann–Whitney. ^*∗∗*^ Chi-squared test. ^*∗∗∗*^Independent Student's *t*-test.

**Table 2:** Hospital Anxiety and Depression Scale (HADS) total and subscales scores between control and experimental groups of ICU patients´ relatives.

		Control Mean ± SD (IL-SL, 95% CI)	Experimental Mean ± SD (IL-SL, 95% CI)	Control vs experimental *p* value
Anxiety	Before ICU admission	8.84 ± 5.11 6.54–11.14	8.52 ± 4.78 6.37–10.67	0.422
	3 days after ICU discharge	6.78 ± 5.60 4.26–9.30	7.42 ± 4.98 5.18–9.66	0.408
	90 days after ICU discharge	5.26 ± 4.75 3.12–7.40	6.47 ± 5.20 4.13–8.81	0.408
Depression	Before ICU admission	5.05 ± 4.23 3.14–6.95	4.73 ± 4.09 2.89–6.57	0.357
	3 days after ICU discharge	5.42 ± 4.40 3.44–7.39	5.94 ± 5.03 3.68–8.21	0.366
	90 days after ICU discharge	1.73 ± 4.10 0.11–3.58	2.94 ± 5.66 0.40–5.49	0.349
Total score	Before ICU admission	13.89 ± 8.62 10.01–17.77	13.26 ± 8.00 9.66–16.86	0.229
	3 days after ICU discharge	12.21 ± 9.32 8.01–16.40	13.36 ± 8.97 9.33–17.40	0.227
	90 days after ICU discharge	7.00 ± 7.86 3.46–10.53	9.42 ± 9.73 5.04–13.79	0.202

SD, standard deviation; 95% CI, 95% confidence interval; IL, inferior limit; SL, superior limit; ICU, intensive care unit; HADS, Hospital Anxiety and Depression Scale. A *p* value < 0.05 was considered statistically significant with a 95% CI. Higher HADS scores indicated higher values of anxiety, depression, and total major depression.

**Table 3:** Impact of Event Scale-Revised (IES-R) scores between control and experimental groups of ICU patients´ relatives.

	Control Mean ± SD (IL-SL, 95% CI)	Experimental Mean ± SD (IL-SL, 95% CI)	Control vs experimental *p* value
Before ICU admission	6.10 ± 7.03 2.94–9.26	6.15 ± 7.58 2.74–9.56	0.491

3 days after ICU discharge	15.78 ± 21.45 6.14–25.43	18.15 ± 18.34 9.90–26.40	0.358

90 days after ICU discharge	8.21 ± 13.10 2.31–14.10	13.84 ± 16.08 6.61–21.07	0.122

SD, standard deviation; 95% CI, 95% confidence interval; IL, inferior limit; SL, superior limit; ICU, intensive care unit; IES-R, Impact of Event Scale-Revised. A *p* value < 0.05 was considered statistically significant with a 95% CI. A Higher IES-R score indicated higher values of stress.

**Table 4:** Critical Care Family Needs Inventory (CCFNI) total and subscales scores between control and experimental groups of ICU patients´ relatives.

	Control Mean ± SD (IL-SL, 95% CI)	Experimental Mean ± SD (IL-SL, 95% CI)	Control vs experimental *p* value
Medical care	1.68 ± 1.52 0.99–2.37	1.84 ± 2.14 0.87–2.80	0.397
Communication	1.94 ± 1.98 1.05–2.84	1.78 ± 2.43 0.69–2.88	0.414
Personal attention	2.68 ± 1.79 1.87–3.49	3.36 ± 2.29 2.33–4.39	0.156
Perceived improvements	3.63 ± 1.49 2.95–4.30	3.31 ± 2.05 2.39–4.24	0.295
Total score	9.94 ± 3.65 8.30–11.59	10.31 ± 5.85 7.68–12.94	0.408

SD, standard deviation; 95% CI, 95% confidence interval; IL, inferior limit; SL, superior limit; ICU, intensive care unit; CCFNI, Critical Care Family Needs Inventory. A *p* value < 0.05 was considered statistically significant with a 95% CI. A higher CCFNI score indicated higher satisfaction with the received attention.

**Table 5:** Family Satisfaction with Care in the Intensive Care Unit (FS-ICU) total, subscales, and items scores between control and experimental groups of ICU patients´ relatives.

	Control Mean ± SD (IL-SL, 95% CI)	Experimental Mean ± SD (IL-SL, 95% CI)	Control vs experimental *p* value
ITEM 1-1	1.63 ± 0.76	1.52 ± 0.90	0.350
Concern and care of the ICU staff	1.28–1.97	1.11–1.93	
ITEM 1-2	1.78 ± 1.03	1.52 ± 0.84	0.197
Symptom management: pain	1.32–2.25	1.14–1.90	
ITEM 1–3	2.63 ± 1.89	2.52 ± 1.71	0.429
Symptom management: dyspnea	1.78–3.48	1.75–3.29	
ITEM 1–4	2.47 ± 1.80	2.68 ± 1.73	0.358
Symptom management: agitation	1.66–3.28	1.90–3.46	
ITEM 1–5	1.57 ± 1.01	1.84 ± 1.21	0.236
Consideration of your needs	1.12–2.03	1.29–2.38	
ITEM 1–6	1.89 ± 1.14	2.78 ± 1.43	0.020
Emotional support	1.37–2.41	2.14–3.43	
ITEM 1–7	1.84 ± 1.34	1.78 ± 1.18	0.449
Care coordination	1.23–2.44	1.25–2.32	
ITEM 1–8	1.78 ± 1.03	2.05 ± 1.31	0.248
Concern and care of the ICU staff	1.32–2.25	1.46–2.64	
ITEM 1–9	1.52 ± 0.96	1.52 ± 0.84	0.500
Skills and competence of ICU nurses	1.09–1.95	1.14–1.90	
ITEM 1–10	2.21 ± 1.31	1.84 ± 1.16	0.183
Frequency of communication with nurses	1.61–2.80	1.31–2.36	
ITEM 1–11	1.63 ± 1.25	1.31 ± 0.67	0.170
Skill and competence of ICU doctors	1.06–2.19	1.01–1.61	
ITEM 1–12	1.78 ± 0.91	1.89 ± 1.10	0.375
ICU environment	1.37–2.20	1.40–2.38	
ITEM 1–13	2.94 ± 0.77	3.10 ± 1.14	0.311
Waiting room environment	2.59–3.29	2.58–3.62	
ITEM 1–14	3.15 ± 1.67	4.05 ± 1.26	0.035
Satisfaction with the care provided by the patients' relatives	2.40–3.91	3.48–4.62	
1^st^ part total score	28.89 ± 11.45	30.47 ± 9.50	0.323
Satisfaction with healthcare	23.74–34.04	26.20–34.74	
ITEM 2-1	1.84 ± 1.01	2.05 ± 1.12	0.274
Frequency of communication with ICU doctors	1.38–2.29	1.54–2.56	
ITEM 2-2	1.94 ± 0.84	1.52 ± 0.69	0.051
Ease of getting information	1.56–2.32	1.21–1.83	
ITEM 2-3	2.10 ± 1.19	1.63 ± 0.89	0.087
Understanding of the information	1.56–2.64	1.22–2.03	
ITEM 2–4	2.15 ± 1.11	1.68 ± 1.05	0.094
Honest information	1.65–2.66	1.20–2.15	
ITEM 2–5	2.63 ± 1.49	2.00 ± 1.20	0.080
Accurateness of information	1.95–3.30	1.45–2.54	
ITEM 2–6	2.57 ± 1.53	1.89 ± 1.14	0.064
Consistency of information	1.88–3.27	1.37–2.41	
ITEM 2–7	3.84 ± 1.01	3.31 ± 1.49	0.105
Feeling of exclusion in the decision-making	3.38–4.29	2.64–3.98	
ITEM 2–8	3.84 ± 0.89	3.52 ± 1.12	0.172
Feeling of support in the decision-making	3.43–4.24	3.02–4.03	
ITEM 2–9	3.78 ± 0.85	3.15 ± 1.57	0.066
Feeling of control on the care of your relative	3.40–4.17	2.45–3.86	
ITEM 2–10	2.21 ± 0.53	1.73 ± 0.56	0.005
Adequate time to raise your concerns and answer your questions	1.96–2.45	1.48–1.98	
ITEM 2–11	0.00 ± 0.00	0.00 ± 0.00	1.000
In case of patient's death: prolongation of life	0.00–0.00	0.00–0.00	
ITEM 2–12	0.00 ± 0.00	0.00 ± 0.00	1.000
In case of patient's death: last hours of life	0.00–0.00	0.00–0.00	
ITEM 2–13	0.00 ± 0.00	0.00 ± 0.00	1.000
In case of patient´s death: staff support	0.00–0.00	0.00–0.00	
2^nd^ part total score	26.94 ± 5.16	22.52 ± 4.11	0.003
Family satisfaction with decision-making	24.62–29.27	20.67–24.37	
General total score	55.84 ± 15.35 48.93–62.74	53.00 ± 10.08 48.46–57.53	0.252

SD, standard deviation; 95% CI, 95% confidence interval; IL, inferior limit; SL, superior limit; ICU, intensive care unit; FS-ICU, Family Satisfaction with Care in the Intensive Care Unit. A *p* value < 0.05 was considered statistically significant with a 95% CI. A Higher FS-ICU score indicated higher satisfaction with the received attention.

**Table 6:** Family Satisfaction with Care in the Intensive Care Unit (FS-ICU) dichotomous items responses between control and experimental groups of ICU patients´ relatives.

	Control Frequency (%)	Experimental Frequency (%)	Control vs experimental *p* value
Lack of time in medical information			
Yes No	0 (0%) 19 (100%)	2 (10.5%) 17 (89.5%)	0.146
Need to improve the information room			
Yes No	2 (10.5%) 17 (89.5%)	1 (5.3%) 18 (94.7%)	0.547
Excessive noise in the ICU			
Yes No	0 (0%) 19 (100%)	1 (5.3%) 18 (94.7%)	0.311
Appreciation for the received healthcare			
Yes No	5 (26.3%) 14 (73.7%)	9 (47.4%) 10 (52.6%)	0.179
Lack of entertainment for patients			
Yes No	1 (5.3%) 18 (94.7%)	0 (0%) 19 (100%)	0.311
Dirty rooms			
Yes No	0 (0%) 19 (100%)	2 (10.5%) 17 (89.5%)	0.146
Lack of staff			
Yes No	2 (10.5%) 17 (89.5%)	2 (10.5%) 17 (89.5%)	1.000
Increase in visiting time			
Yes No	1 (5.3%) 18 (94.7%)	1 (5.3%) 18 (94.7%)	1.000
Toilet areas for family members			
Yes No	1 (5.3%) 18 (94.7%)	3 (15.8%) 16 (84.2%)	0.290
Rooms and lockers for family members			
Yes No	1 (5.3%) 18 (94.7%)	3 (15.8%) 16 (84.2%)	0.290
Poor regulation of environmental temperature			
Yes No	0 (0%) 19 (100%)	0 (0%) 19 (100%)	1.000
Lack of religious support			
Yes No	0 (0%) 19 (100%)	1 (5.3%) 18 (94.7%)	0.311

ICU, intensive care unit; FS-ICU, Family Satisfaction with Care in the Intensive Care Unit. A *p* value < 0.05 was considered statistically significant with a 95% confidence interval.

## Data Availability

Raw data will be available upon request to the corresponding author.
